# Characterization of Grapevine Fanleaf Virus Isolates in ‘Chardonnay’ Vines Exhibiting Severe and Mild Symptoms in Two Vineyards

**DOI:** 10.3390/v14102303

**Published:** 2022-10-20

**Authors:** Julie Kubina, Jean-Michel Hily, Pierre Mustin, Véronique Komar, Shahinez Garcia, Isabelle Rachel Martin, Nils Poulicard, Amandine Velt, Véronique Bonnet, Laurence Mercier, Olivier Lemaire, Emmanuelle Vigne

**Affiliations:** 1 INRAE, SVQV UMR-A 1131, Université de Strasbourg, 68000 Colmar, France; 2 IFV, 30240 Le Grau-Du-Roi, France; 3 LVBE, UPR 3991, Université de Haute-Alsace, 68000 Colmar, France; 4 PHIM, Université Montpellier, IRD, INRAE, Cirad, SupAgro, 34000 Montpellier, France; 5 Maison Moët & Chandon, 20 Avenue de Champagne, 51200 Épernay, France

**Keywords:** grapevine fanleaf virus, fanleaf degeneration, grapevine, symptomatology, mild isolates, cross-protection, genetic and phenotypic diversity, high throughput sequencing, virome

## Abstract

Fanleaf degeneration is a complex viral disease of *Vitis* spp. that detrimentally impacts fruit yield and reduces the productive lifespan of most vineyards worldwide. In France, its main causal agent is grapevine fanleaf virus (GFLV). In the past, field experiments were conducted to explore cross-protection as a management strategy of fanleaf degeneration, but results were unsatisfactory because the mild virus strain negatively impacted fruit yield. In order to select new mild GFLV isolates, we examined two old ‘Chardonnay’ parcels harbouring vines with distinct phenotypes. Symptoms and agronomic performances were monitored over the four-year study on 21 individual vines that were classified into three categories: asymptomatic GFLV-free vines, GFLV-infected vines severely diseased and GFLV-infected vines displaying mild symptoms. The complete coding genomic sequences of GFLV isolates in infected vines was determined by high-throughput sequencing. Most grapevines were infected with multiple genetically divergent variants. While no specific molecular features were apparent for GFLV isolates from vines displaying mild symptoms, a genetic differentiation of GFLV populations depending on the vineyard parcel was observed. The mild symptomatic grapevines identified during this study were established in a greenhouse to recover GFLV variants of potential interest for cross-protection studies.

## 1. Introduction

Grapevine is a crop hosting many viruses (more than 90 viruses and viroids have been identified in grapevines) with some of them affecting its cultivation and fruit production worldwide [1,2,3]. Among grapevine viral diseases, fanleaf degeneration is the most destructive [4]. In France, this disease is estimated to be present over more than 60% of the vineyard hectarage [5]. The main virus responsible for fanleaf degeneration is grapevine fanleaf virus (GFLV), a soil-borne nepovirus of the *Secoviridae* family, specifically transmitted by the ectoparasitic dagger nematode *Xiphinema index* [6]. GFLV infections can cause massive crop losses (up to 80%) and a shorter productive lifespan of vineyards due to a progressive degeneration that can lead to vine death [4,7,8,9].

Typical symptoms associated with fanleaf degeneration are foliar discolouration (i.e., complete yellowing, vein banding, mosaic), foliar deformations (i.e., small leaf, leaf with open petiole sinus), shoot abnormalities (i.e., short internodes, fasciation) and/or growth inhibition of the plant (i.e., stunting) [6]. These symptoms can vary in intensity and range from mild to severe, depending on environmental factors, rootstock genotype, *Vitis vinifera* cultivar (cv.), viral strain, vineyard site, and viticultural practices [10,11,12,13]. While viral determinants of specific disease symptoms have been identified in herbaceous model plants [14,15,16], they have yet to be determined in the main natural host of GFLV, grapevine.

The genome of GFLV is composed of two positive-sense RNAs named RNA1 and RNA2 of 7.3 and 3.8 kb in size, respectively [6]. Both RNAs are necessary for infection in planta, and both contain a single open reading frame (ORF1 and ORF2) that codes for a polyprotein (P1 and P2) processed by the viral proteinase into functional proteins. A recent genetic diversity study based on a cohort of 40 GFLV ORF1 and 80 ORF2 sequences from around the world described a very high degree of polymorphism for both ORFs with 0.127 and 0.130 nucleotide substitutions per site, respectively [17]. Many recombination events were identified all along the GFLV genome, potentially explained by the presence of many variants within a single plant [17,18,19,20]. An additional satellite RNA (RNA3) of 1.1 kb is associated with some GFLV isolates [21]. This third RNA contains a unique ORF (ORF3) coding for a non-structural protein of unknown function and does not seem to interfere with viral pathogenicity [22].

Current strategies to control fanleaf degeneration rely on prophylactic measures based on the production of certified materials (i.e., free of GFLV and other damaging viruses) which are effective in avoiding the introduction of the virus in uninfected vineyards [23]. However, once the fanleaf pathosystem (viruliferous nematodes) is established in a vineyard it is almost impossible to eradicate [24]. The removal of infected plants followed by a five to seven years fallow period decreases the nematode vector population, but this option is economically unpractical for grape growers [23]. Rootstocks tolerant to *X. index* are available but they seem to only delay the infection by GFLV [25,26,27]. The use of chemicals to control nematode vector populations has been banned in Europe due to environmental and human health concerns [28,29]; the use of plants exhibiting negative impact on nematodes are currently under study [30] with particular interest of the *Fabaceae* family exhibiting nematicidal activity [31]. Biotechnological approaches to engineer resistance against GFLV are promising but are unlikely to be adopted in Europe [32]. A recessive factor of resistance against GFLV was recently described in *V. vinifera* cv. Riesling, providing interesting perspectives for fanleaf management [33]. However, at least two decades of research will be needed for characterizing this resistance factor, its sustainability, and introducing this genetic trait in grapevine genotypes prior to the deployment of new resistant hybrids in vineyards. For all these reasons, other strategies to mitigate the effects of fanleaf degeneration are needed, particularly in old high-value vineyards where growing vines is very challenging due to the severity of the disease.

In this context, cross-protection offers an interesting biocontrol alternative to the aforementioned strategies for the management of fanleaf degeneration [34]. Cross-protection is a natural phenomenon, first described by McKinney almost a century ago [35], in which a primary infection with a mild virus strain protects a plant from the disease induced by related variants of the same virus [36]. For viral disease management in crops, cross-protection relies on the selection of virus strains that are able to (1) cause mild symptomatic infections while (2) conferring protection against the effects of severe variants [34,37]. In other words, a plant can be deliberately pre-immunized with a mild viral variant to protect it from aggressive counterparts of genetically related variants that will challenge it in the field. Many efforts have been made to explain the protection in primarily infected plants; however, the molecular mechanism(s) underlying cross-protection remain(s) largely unclear [34,38].

Cross-protection is used to control some viruses in different crops of economic importance such as pepino mosaic virus (PepMV) in tomato [39,40] or citrus tristeza virus (CTV) in citrus [41,42]. Mild viral strains are obtained either by laboratory methods, including single local lesion isolations on plant hosts, temperature treatments, physical or chemical treatments, site-directed mutagenesis, or via field selections [34,43,44,45,46,47,48]. In the latter case, mild viral strains were selected in severely diseased fields in which infected plants showed little to no symptom [34,44]. The main advantages of such naturally occurring mild isolates are that: (1) they are well adapted to the cultivar and its environmental conditions and (2) they are expected to ensure protection against local (or endemic) severe variants. A prime example of these mild virus isolate features is the CTV cross-protective mild isolate IAC obtained from sweet orange cv. Pera in Brazil (PIAC isolate). Millions of protected ‘Pera’ trees established in sweet orange orchards show a durable protection against stem pitting strains of CTV, leading to the success of the Brazilian citriculture [42]. Yet, this mild isolate was not efficient on cvs. Hamlin or Valencia, and not protective in all Brazilian regions. It appears that a fundamental requirement to achieve successful cross-protection, at least for this viral species, might be the selection of mild isolates for each cultivar associated with location/locality of interest [44,49,50].

The evaluation of cross-protection to mitigate the impact of GFLV was initiated over thirty years ago in our laboratory with one mild GFLV strain, named GHu, isolated from *V. vinifera* cv. Gloria Hungariae. This strain, which causes mild symptoms in *Chenopodium quinoa*, could prevent under certain inoculation conditions, the development of the severe symptoms induced by the GFLV-F13 strain [51]. Primary-infected vines (cv. Gewurztraminer) with GFLV-GHu exhibited a significantly reduced superinfection rate in a GFLV-infected vineyard site located in the Alsace region of France [52]. However, these cross-protected vines showed reduction of fruit yield over the eight-year study in comparison with non-cross-protected vines, making cross-protection based on GHu strain of limited interest under these conditions [52].

Thus, to pursue cross-protection as an effective biocontrol management strategy against fanleaf degeneration, it is fundamental to identify new mild GFLV strains in diseased vineyards, especially in the Champagne region of France where fanleaf degeneration is one of the major concerns of wine grape growers [53]. The main objective of this study was to identify GFLV-infected vines showing mild symptoms in two heavily diseased vineyard parcels and to characterize the diversity of GFLV isolates in infected ‘Chardonnay’ vines by high-throughput sequencing (HTS). By recording qualitative and quantitative phenotypic traits during four consecutive years, four mildly symptomatic vines infected with potentially mild GFLV isolates were identified.

## 2. Materials and Methods

### 2.1. Vineyard Sites

Two commercial GFLV-infected vineyard sites, one in Chouilly (named ‘Pa’) and the other in Cramant (named ‘Py’), two villages spaced 5.4 km apart in the Champagne region of France, were selected for this study. These two vineyard parcels were planted in the 1980s after treatment with a systemic herbicide and soil disinfection using nematicidal chemicals (Shell DD and Temik10G). In line with viticultural practices in the Côte des blancs area in the Champagne region, vines consisted of *V. vinifera* cv. Chardonnay grafted onto a rootstock, likely 41B (*V. berlandieri* × *V. riparia*), as indicated by wine growers of both sites.

### 2.2. Disease Symptom Monitoring, Grapevine Leaf Sampling, and GFLV Detection

From 2016 to 2019, qualitative and quantitative phenotypic traits were recorded on selected vines in the two above mentioned ‘Chardonnay’ vineyard parcels (Figure S1). Qualitative phenotypic traits belonging to three kinds of symptoms (discolouration of leaves, deformation of leaves and stunting of plants) were evaluated and recorded every year in June, the best time to visualize GFLV symptoms in the northern hemisphere. The same symptom scoring as described in our recent article was used [13]: discolouration and deformations of leaves were estimated on individual vines and scored from 0 to 4 (0: absence of symptom, 1: 1 to 25%, 2: 26–50%, 3: 51–75%, and 4: 76–100% of symptomatic leaves) (Figure S2). Similarly, vine stunting was assessed and scored from 0 to 4 (0: no stunting, 1: low, 2: medium, 3: strong, 4: very strong stunting). An overall symptom severity score was determined by summing all qualitative trait scores measured for each plant. Quantitative phenotypic traits were also assessed on individual vines by counting the number of grape clusters at harvest and by measuring pruning wood weights in winter.

Five to six young apical leaves from different shoots were collected in the spring for every plant from 2016 to 2019 and stored frozen prior to analyses [19]. The presence of GFLV was evaluated by double antibody sandwich-enzyme linked immunosorbent assay (DAS-ELISA) using GFLV specific antibodies (BIOREBA AG, Reinach, Switzerland), and by molecular techniques as previously described [19].

### 2.3. Statistical Analyses of Qualitative and Quantitative Phenotypic Traits

The significances of the differences between symptom categories for all traits were tested by comparison of means (Student’s *t* test or Welch’s *t* test) or medians (Kruskal–Wallis *H* test) and completed by Tukey’s honestly significant difference (HSD) tests when needed. The relationship between the overall symptom scores and fruit yields was analysed using Spearman’s rank correlation coefficient. Principle component analysis (PCA) was performed to evaluate associations among individuals and among the variables using the FactoMineR package [54]. Differences were considered significant when *p* < 0.05. Boxplots were drawn to show the median (horizontal bold line) and interquartile range with lonely dots representing extreme data. All data and graphic representations were performed using R software (version 4.0.2; R Core Team, 2021, https://www.r-project.org/ (accessed on 7 April 2021)). Raw data are available in Table S1.

### 2.4. RNA Sequencing and Downstream Bioinformatics Analyses

Total RNA extractions (from grapevine leaves collected in 2017), cDNA library preparations, Illumina sequencing and HTS data analyses (for the determination of the viral status) of the twenty vines selected for this study are described in our previous article [19]. Raw Illumina sequence data files are available on ENA-EBI, project N°PRJEB54883. For vine Py43, total RNA extracted from leaves collected in 2018 was sequenced through a small RNA-Seq run on a Hiseq Instrument at 1 × 50 pb and multiplexed at Fasteris (Plan-les-Ouates, Switzerland).

### 2.5. GFLV Sequences Analyses, Genetic Diversity and Recombination Detection

Each GFLV consensus sequence generated by de novo assembly [55] was named as follows: first by indicating the geographic origin of the sample [Pa] or [Py] and the identification number of the vine, second by designating the genomic or satellite GFLV RNA under consideration [1,2] or [3] and third by classifying the molecular variant depending on its accumulation based on the number of reads per kilobase per million of total reads (RPKM), the first being the one with the highest value.

GFLV consensus sequences were aligned using CLUSTALW (codon-based multiple alignments) with default parameters in MEGA7 software [56]. Maximum likelihood (ML) phylogenetic trees were reconstructed using the same software. The best-fit ML model for each sequence alignment was used (GTR + G + I, GTR + G, T92 + G for the complete ORF1, ORF2, ORF3 datasets, respectively) and bootstrapping analyses of 100 replicates were performed. BioEdit software [57] was employed to construct nucleotide identity matrices for clade definition. Trees were visualized and fashioned in FigTree v1.3.1. (Institute of Evolutionary Biology, University of Edinburgh, Edinburgh. http://tree.bio.ed.ac.uk/software/figtree/ (accessed on 27 August 2019)).

Genetic diversity (π) values were calculated in MEGA7 software using the best-fit ML model determined for each dataset (TN93 + G for ORF1, ORF2, 1B^Hel^ and 1E^Pol^; K2 + G for 1A, 1C^VPg^, 2A and 2B^MP^; T92 + G for 1D^Pro^ and 2C^CP^) and bootstrapping analyses of 100 replicates were performed. π and Tajima’s D (*D_T_*) sliding window analyses were conducted in DnaSP v6.12.03 [58] with window length of 100 nts and step size of 25 nts parameters. The difference between non-synonymous (*dN*) and synonymous (*dS*) substitutions over the coding sequences of GFLV populations was estimated by the Kumar method in MEGA7 software. GFLV populations were compared by calculating the fixation index (*F_ST_*) in Arlequin v3.5.2.2 [59] using the above-mentioned models. Significance of the differences was obtained by performing 1000 permutations. Finally, potential recombination events were estimated by using RDP4 and the default settings of the seven algorithms: RDP, GENECONV, BootScan, MaxChi, Chimaera, SiScan and 3Seq [60]. Only recombination events detected by five or more methods were considered.

## 3. Results

### 3.1. Identification of ‘Chardonnay’ Vines Showing Distinct Fanleaf Symptoms

To select potentially protective GFLV isolates suitable for cross-protection of ‘Chardonnay’ vines in the Champagne region of France, our strategy was to prospect old vineyards highly impacted by fanleaf degeneration disease and identify vines displaying mild disease symptoms. Two 30-year-old ‘Chardonnay’ parcels, ‘Py’ and ‘Pa’, with an estimated vine mortality of 15 to 20% very likely due to GFLV infections, were selected for this study in the ‘Côte des Blancs’ area. Despite soil disinfection in these parcels in the 1980s, propagation of fanleaf degeneration was inexorable with increasing symptomatic patches (or areas) being observed yearly. The presence of the nematode vector *X. index* was confirmed in both parcels after soil analysis (data not shown).

In spring 2016, we surveyed both sites for vigorous vines with a good potential for fruit production and proximal to severely diseased vines. An overall symptom score achieved by the addition of the scores for three qualitative phenotypic traits, i.e., leaf discolouration, leaf deformations and stunting of the plants, was used to classify the vines (Figure S2). Mildly symptomatic and asymptomatic vines were considered of potential interest for our study if they received a maximum of ‘3′ for the overall symptom score. Five mild (‘M’) symptomatic and five severe (‘S’) symptomatic vines were selected in each vineyard site and all these vines were around 30 years old (Table S1). In summer and winter 2016, quantitative traits, i.e., cluster number, fruit yield, fruit yield per cluster, cane number, pruning wood weight per vine and pruning wood weight per cane were assessed for the ten selected vines at each vineyard site. An additional vine named Py43 was incorporated in the M class in September 2016 (Figure S1, Table S1). Therefore, our plant population under study consisted of 21 vines, including 11 and 10 vines grouped into the M and S classes, respectively. A comparative analysis of qualitative phenotypic trait scores and quantitative data for each class revealed significant differences for all the comparisons, except for leaf deformation and cane number (Figure S3, Table S2).

The presence of GFLV in leaves of the selected vines was tested by DAS-ELISA. While all selected grapevines were located in severely diseased areas, the virus was surprisingly only detected in less than half of the grapevines displaying mild symptoms (Table 1). These results were confirmed by using several diagnostic molecular techniques, as previously reported [19]. However, the virus was detected in all the severely symptomatic vines. Consequently, the cohort of the selected grapevines was divided in three categories (Table 1 and Table S1): ‘M−’ (mild symptomatic GFLV-free vines), ‘M+’ (mild symptomatic GFLV-infected vines) and ‘S+’ (severe symptomatic GFLV-infected vines). These three categories were used to examine the candidates for cross-protection by comparing them to either non-infected vines or severely infected ones.

**Figure 1:**
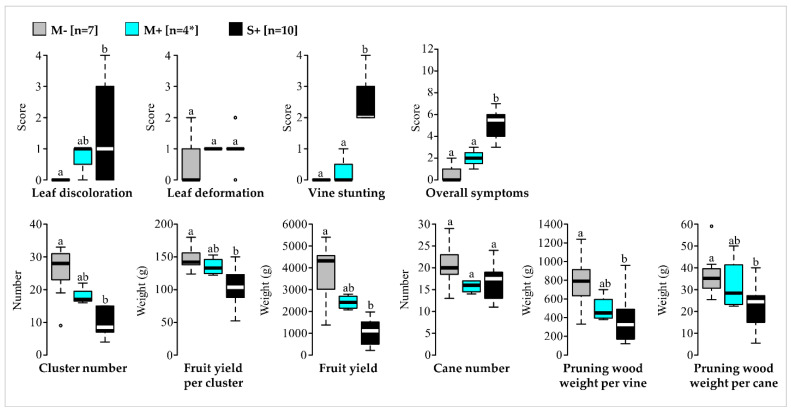
Comparative analyses of qualitative and quantitative traits for the three vine categories in 2016 (M−, mild symptomatic GFLV-free vines (in grey); M+, mild symptomatic vines infected with GFLV (in cyan) and S+, severe symptomatic vines infected with GFLV (in black)). Number of vines (n) are given in brackets. * For the vines in the M+ category, only three vines were considered in June 2016 for the evaluation of qualitative traits (leaf discolouration, leaf deformation, vine stunting, overall symptoms). Significance was tested with Student’s *t*, Welch’s *t* or Kruskal–Wallis *H* tests. Boxplots show the median (horizontal bold line) and the interquartile range with lonely dots representing extreme data. Different letters (a,b) indicate significant differences (*p* ≤ 0.05). Raw data and results of the statistical tests are available in Tables S1 and S3.

Comparisons of phenotypic traits showed that scores for vines in the M+ category ranged generally between scores for vines in the M− and S+ categories (Figure 1). There are no significant differences in any traits between M− and M+ categories, unlike M− and S+ which were different for most traits (Figure 1, Table S3). Thereby four mild symptomatic GFLV-infected ‘Chardonnay’ vines (Pa1, Py18, Py19 and Py43) were identified in 2016 and were considered as candidates hosting potentially mild isolates of interest for cross-protection.

### 3.2. Phenotypic Differentiation and Overtime Stability of M−, M+ and S+ Categories

To assess the phenotypic durability of the three vines categories defined in 2016, qualitative and quantitative traits were monitored from 2017 to 2019 and the presence of GFLV was evaluated by DAS-ELISA (Table 1 and Table S1). GFLV was always detected in leaves from M+ and S+ vines, while remaining undetectable by DAS-ELISA in leaves from vines in the M− category (Table 1).

**Figure 2:**
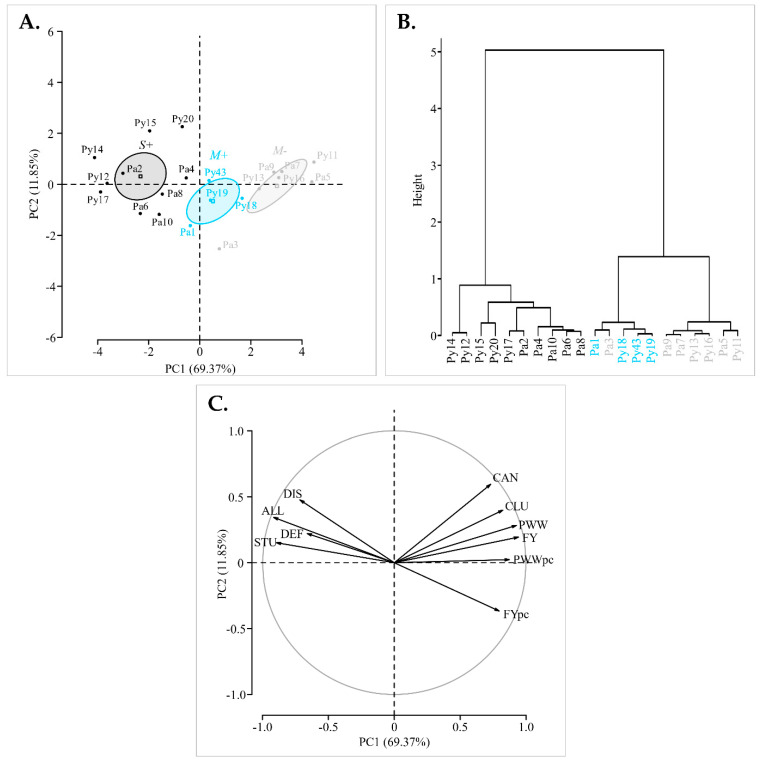
Principal components analysis (PCA) and hierarchical clustering of 21 vines according to their mean symptom scores and yield values over the four-year study (from 2016 to 2019). (**A**) Biplot representing the ‘M−’ (grey), ‘M+’ (cyan) and ‘S+’ (black) individuals. Projection of M−, M+ and S+ categories are represented by squares with their confidence ellipses (95%). (**B**) Dendrogram representing the hierarchical clustering based on the individuals coordinates on the 10 principal components. (**C**) Loading biplot, the vectors show the relative importance of each variable in discriminating amongst the observations. Longer vectors indicate greater contribution. Vectors point in the direction they ordinate the observations (DIS: Leaf discolouration, DEF: leaf deformation, STU: vine stunting, ALL: overall, CLU: cluster number, FY: fruit yield, FYpc: fruit yield per cluster, CAN: cane number, PWW: pruning wood weight per vine, PWWpc: pruning wood weight per cane). Results of the Fisher’s test are available in Table S4.

Mean symptom scores and mean yield values from 2016 to 2019 for each individual vine were assessed by PCA and hierarchical clustering on principle components. The representation accuracy is supported by the two first principal components explaining 81.22% of the individuals and variables cloud variances (Figure 2).

The individuals were clearly separated along the first principal component (PC1) according to their categories (M−, M+ and S+). Fisher’s tests, assessing correlations between both symptom and yield variables, and PC1, showed respectively significant negative and positive correlation coefficients (Table S4). Thus, PC1 ordinates the S+ vines with severe symptoms and low yields in one direction (to the left), and the M− vines, showing mild symptoms and higher yields in the opposite direction (to the right). M+ vines were dispersed in the middle of the two groupings (Figure 2). Furthermore, the dendrogram resulting from hierarchical clustering on individuals coordinates on all principal components (i.e., explaining 100% of the variability) confirmed this trend with three major clusters with an almost perfect segregation of M−, M+ and S+ vines (Figure 2). It should be noted that overall symptom score and fruit yield were the main indicators of the variability with the longer vector values, indicating their greater contribution in the separation of the data (Figure 2). Consequently, results obtained with these two variables were further evaluated in our study, while the other phenotypic traits are only detailed in Supplementary Data.

**Figure 3:**
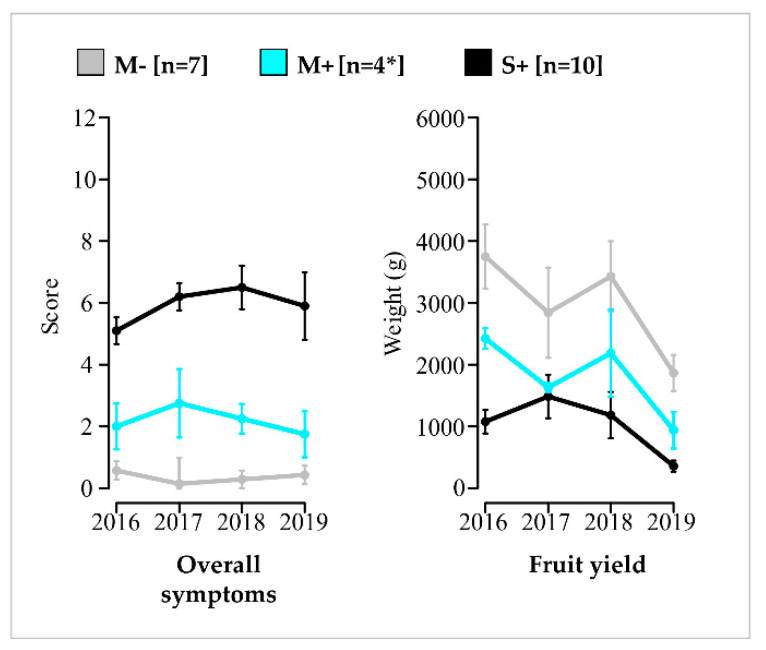
Evolution of yearly mean overall symptom scores and fruit yields of vines in the M−, M+ and S+ categories and their standard error. Results for vines in the M− (in grey), M+ (in cyan) and S+ (in black) categories are shown. Number of vines (n) is given in brackets. Means values are connected by bold lines and standard errors are materialized by vertical bars. * For the M+ category, only three vines were considered in June 2016 for the evaluation of the qualitative traits.

Regarding phenotypic traits evolution from 2016 to 2019, the annual mean values of M+ vines ranged each year between values obtained for vines in the M− and S+ categories, illustrating a steady pattern. This trend was observed for overall symptoms, fruit yield (Figure 3) and other traits (Figure S4). Therefore, each category of vines remains constant along the study.

**Figure 4:**
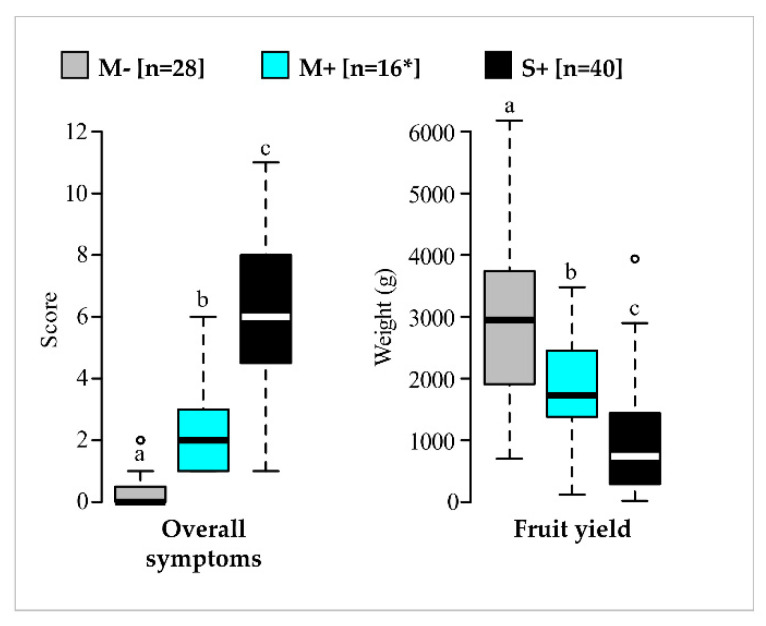
Comparison of overall symptom scores and fruit yields over four years for vines in M−, M+ and S+ categories. Results for vines in the M− (in grey), M+ (in cyan) and S+ (in black) categories are shown. Boxplots show the median (horizontal bold line) and the interquartile range with lonely dots representing extreme data. Different letters (a,b,c) indicate significant differences (*p* ≤ 0.05). Raw data and results of the statistical tests are available in Tables S1 and S3, respectively. Number of vines (n) is given in brackets. * For the M+ category, 15 replicates were considered for overall symptom scores.

Across the four-year study, statistical differentiation of the three categories of vines was observed for the two main indicators (Figure 4, Table S3). In particular, vines in the M+ category showed significantly lower overall symptom scores and higher fruit yields in comparison to vines in the S+ category. Similar trends were observed with the other variables, even though differences were not always statistically validated (Figure S5, Table S3). For fruit production, vines in the M+ category (mean 1795 g/vine with 95% confidence interval at 1305 to 2284) produced about 40% less relatively to vines in the M− category (mean 2971 g/vine with 95% confidence interval at 2471 to 3471). However, the same M+ category was 82% more productive than the S+ category (mean 988 g/vine with 95% confidence interval between 697 and 1279). The promising results of the M+ category underline the beneficial agronomic performances provided by mildly symptomatic GFLV-infected vines in comparison to severely diseased ones, thus confirming their potential use in a cross-protection program.

### 3.3. Virome Description and Genetic Characterization of GFLV Isolates: Mixed Infections of Highly Divergent Molecular Variants

To characterize the molecular variants in the GFLV-infected vines, HTS was performed using total RNA isolated from leaves collected on the 21 studied grapevines. Besides GFLV, the usual grapevine background virome (i.e., GRSPaV, grapevine rupestris stem pitting-associated virus, HSVd, hop stunt viroid, and GYSVd-1, grapevine yellow speckle viroid 1) was found in all vines (Table S5). Other viruses of the *Tymoviridae* family (GFkV, grapevine fleck virus, GSyV1, grapevine Syrah virus 1, GRVFV, grapevine rupestris vein feathering virus, and GRGV, grapevine red globe virus) were detected in some plants. Grapevine Pinot gris virus (GPGV) was identified in a single vine and grapevine leafroll-associated virus 2 (GLRaV-2,) was detected in four of them (Table S5). Regarding the accumulation of these viruses, viral normalized read counts was low (1 to 219 RPKM) in comparison to GFLV genomic (362 to 3706 RPKM) and satellite (1145 to 6809 RPKM) RNAs (Table S5).

Consensus sequences of GFLV RNAs that were assembled through HTS analyses were considered as distinct molecules and referred to as molecular variants. Partial 5′ untranslated regions (UTR) and 3′UTR were recovered for these molecular variants, but de novo assembly was challenging in these regions due to highly conserved stem loops shared between GFLV RNAs. Therefore, due to this limitation, we focused subsequent analyses on the GFLV ORF sequences only. In total, 27 GFLV ORF1, 19 GFLV ORF2 and five GFLV satellite ORF3 molecular variants (GenBank ON991744-ON991794) were obtained after de novo sequence assembly (Table S5).

The length of ORF1 (6852 nts), ORF2 (3330 nts), and ORF3 (1023 nts) was identical for all the molecular variants characterized in this study, except for Py17-3-1 ORF3 molecular variant (1014 nts). Analyses of the nucleotide and deduced protein sequences indicated identities of 86.6–99.8% in nts (91.9–99.8% in amino acids, aa) for ORF1, 87.8–99.6% in nts (94.2–99.5% in aa) for ORF2 and 82.4–99.7% in nts (77.8–99.4% in aa) for ORF3 (Table S6).

Putative recombination events were assessed using RDP4. Fourteen and four recombination events were predicted within GFLV ORF1 and ORF2, respectively, but none in GFLV ORF3 (Figure S6). Recombination breakpoints were identified within all viral genomic coding regions, except for the ones coding for the helicase (1B^Hel^) and the VPg (1C^VPg^).

**Figure 5:**
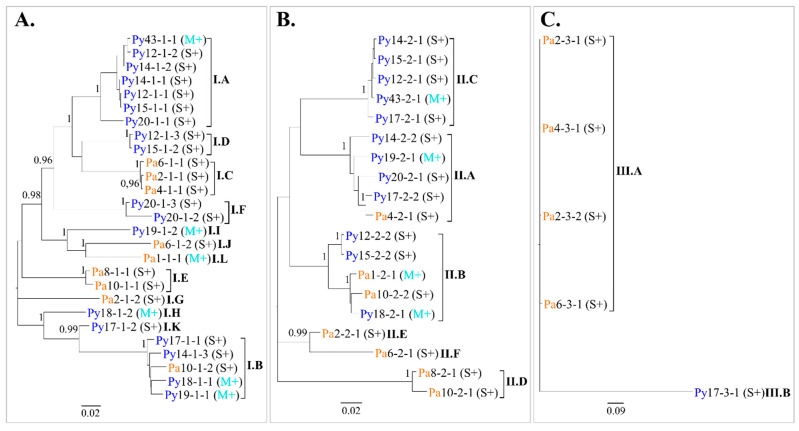
Phylogenetic relationships between grapevine fanleaf virus (GFLV) molecular variants. Maximum likelihood trees were inferred from the complete nucleotide sequences of ORF1 (**A**), ORF2 (**B**) and ORF3 (**C**) recovered from GFLV-infected ‘Chardonnay’ vines. Each sequence name indicates the vineyard site (Pa, in orange and, Py, in deep blue), the sampled vine, the molecular variant and the vine affiliation to a phenotypic category (M+, in cyan and S+, in black) in parenthesis. Clades are named with the Roman numeral corresponding to the GFLV ORF and classified from the most [A] to the least [L] represented clade in sequence number. The names of the distinct clades are highlighted in bold. Sequence variants sharing at least 95% nucleotide identity were considered in the same clade; pairwise nucleotide identities values between GFLV sequences are available in Table S6. Scale bars below each tree show genetic distance. Only bootstrap values ≥ 0.95 are indicated.

A maximum likelihood (ML) tree based on GFLV nucleic acid sequences alignment was constructed for each ORF. Phylogenetic analyses revealed clustering of sequences into twelve, six, and two distinct clades for ORF1, ORF2 and ORF3, respectively (Figure 5). The criteria used to define clade affiliation was at least 95% sequence identity at the nucleotide level (Table S6). Looking at the molecular variant composition in each vine, most of the vines showed mixed GFLV infections with the detection of two divergent variants (i.e., from different clades) of ORF1 in ten vines and of ORF2 in five vines (Table 2). In addition to the numerous variants of each ORFs present within a vine, many different combinations of GFLV ORF1, ORF2 and ORF3 variants belonging to distinct clades were observed. Consequently, every infected vine was unique regarding its composition of GFLV variants, with the exception of two neighbouring vines Py12 and Py15, for which GFLV variants from the same clades (i.e., I.A, I.D, II.B and II.C) were detected (Table 2).

### 3.4. Comparison of GFLV Molecular Variants in the M+ and S+ Categories and in Pa and Py Vineyard Sites

**Figure 6:**
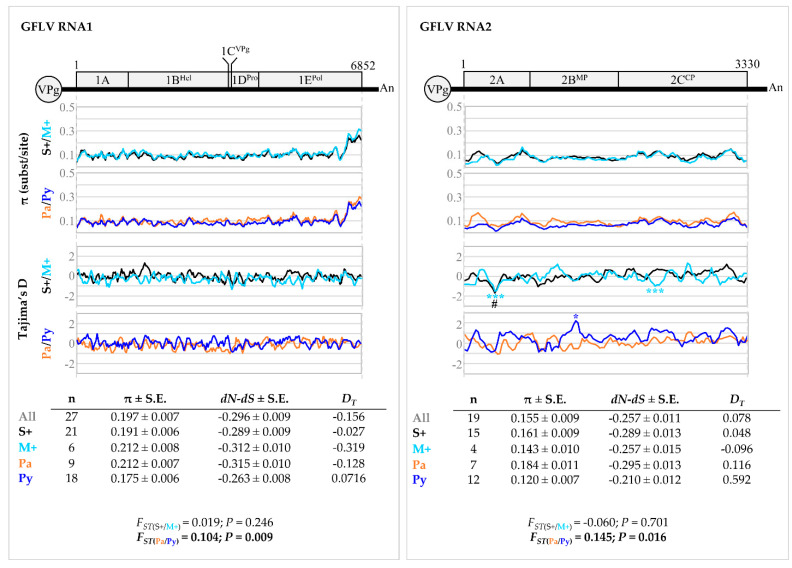
Genetic diversity analyses of grapevine fanleaf virus (GFLV) ORF1 and 2 sequences. Graphics represent genetic diversity (π, substitution per site) along each ORF1 and ORF2 sequence and Tajima’s D (*D_T_*) for evolution study (# *p* < 0.10, * *p* < 0.05, *** *p* < 0.001). Sequences were grouped according to either the phenotypic category or the vineyard site. A colour code was applied for each population: black for ‘S+’, cyan for ‘M+’, orange for ‘Pa’, deep blue for ‘Py’ and dark grey for all sequences. Number of sequences (n), overall genetic diversity π (± standard error, S.E.); the diversity of synonymous (*dS*) and nonsynonymous (*dN*) substitutions (*dN* − *dS* < 0: negative/purifying selection; *dN* − *dS* = 0: neutral/conservative selection; *dN* − *dS* > 0: positive/diversifying selection) and the overall Tajima’s D (*D_T_*) (for all *D_T_* values *p* > 0.10, non-significative) are given in the tables below the graphs. *D_T_* = 0 corresponds to a mutation-drift equilibrium, *D_T_* > 0 indicates balancing selection, sudden population contraction and *D_T_* < 0 distinguishes a recent selective sweep, population expansion after a recent bottleneck. Genetic differentiation of GFLV populations is expressed as the fixation index (*F_ST_*) with associated *p*-value (*p*). Significance (*p* ≤ 0.05) is indicated in bold.

Most phylogenetic clades harboured molecular variants from vines of both M+ and S+ categories. On the other hand, molecular variants from the ‘Pa’ and ‘Py’ vineyards were distributed in different clades with the exception of clades I.B, II.A and II.B (Figure 5). These observations were supported following GFLV populations differentiation and fixation index (*F_ST_*) analysis. Comparison using molecular variant populations in vines in the M+ and S+ categories displayed very low *F_ST_* and non-significant *p*-values (*p* > 0.05), indicating no genetic differentiation between the two populations either when ORF1 (*F_ST_*_(S+/M+)_ = 0.019, *p* = 0.246), ORF2 (*F_ST_*_(S+/M+)_ = −0.060, *p* = 0.701) or any coding regions of either ORFs being considered (Figure 6, Table S7). In contrast, a significant genetic differentiation between molecular variant populations of the ‘Pa’ and ‘Py’ vineyards was statistically validated for both ORF1 and ORF2 sequences (*F_ST_*_(Pa/Py)_ = 0.104, *p* = 0.009 for ORF1; *F_ST_*_(Pa/Py)_ = 0.145, *p* = 0.016 for ORF2), as well as for most coding regions of either ORFs (Figure 6, Table S7). Regarding ORF3 molecular variants, they were only found in four vines out of ten in the S+ category.

Similar nucleotide diversity (π) patterns were found for all molecular variant populations either on ORF1, ORF2 or specific coding regions of these ORFs sequences (Figure 6, Table S7). Regardless of the sequence populations, molecular variants of ORF1 were slightly more diverse than those of ORF2 with overall π values calculated at 0.197 ± 0.007 for ORF1 versus 0.155 ± 0.009 for ORF2. The most divergent region was the 3′ extremity of ORF1 (Figure 6). When looking at the constraints (*dN* − *dS*) acting on ORFs and coding regions, an excess of synonymous versus nonsynonymous substitutions was observed, indicating that these sequences were mainly under negative selection, with the exception of positive values observed for the coding region of 1C^VPg^ (Table S7). This purifying selection trend was essentially the same for all sequence populations analysed. No major differences were observed in the evolution patterns of GFLV molecular variants with all *D_T_* values being negative or very close to 0 for all groups of sequences (*p* > 0.10).

**Figure 7:**
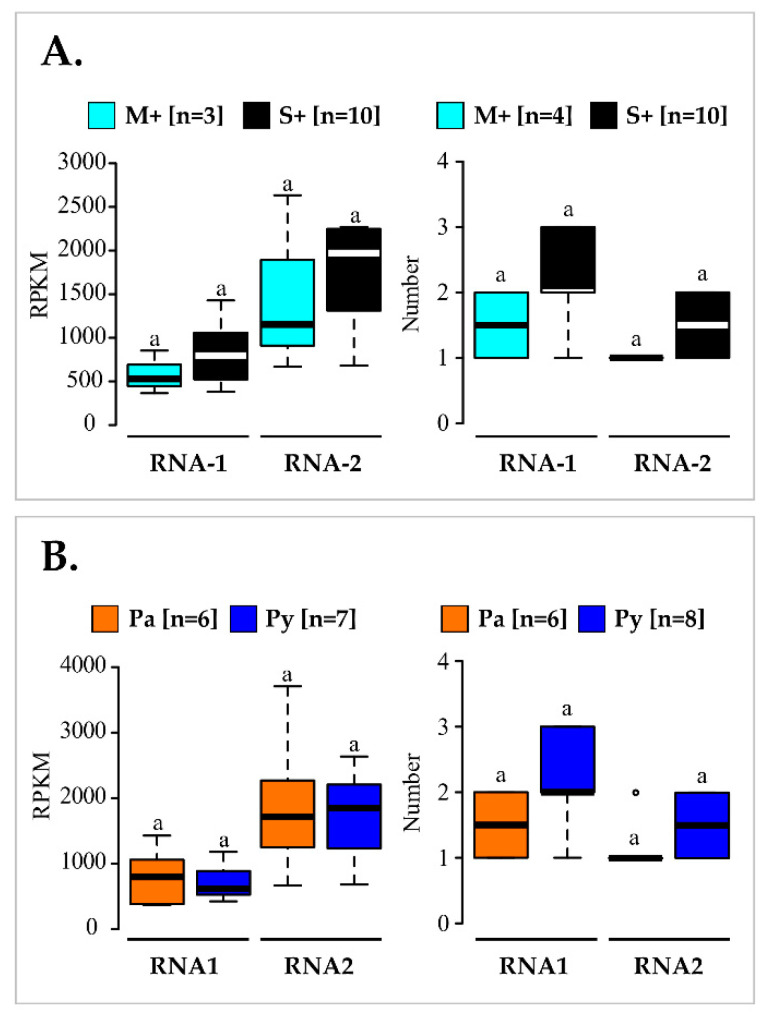
Comparison of grapevine fanleaf virus (GFLV) RNA1 and RNA2 mean RPKM values (left) or mean number of total GFLV genomic RNA variants (right) detected in vines in the M+ versus S+ categories (**A**) or from the Pa versus Py vineyard sites (**B**). Boxplots show the median (horizontal bold line) and the interquartile range with lonely dots representing extreme data. Significance was tested with a Student’s *t* or Kruskal–Wallis *H* tests (for all tests *p* > 0.05, non-significant as indicated by letter ‘a’). Results obtained from vines of the M+ (in cyan) and S+ (in black) categories, and from the Pa (in orange) and Py (in deep blue) are shown. Number of vines (n) is given in brackets. Raw data and results of the statistical tests are available in Tables S5 and S8, respectively.

Finally, an analysis of variance was conducted to compare the viral titre expressed in RPKM deduced from RNA-Seq and the number of variants found in infected vines from either M+/S+ or Pa/Py categories. However, no significant differences (*p* > 0.05) were found (Figure 7, Tables S5 and S8). Thus, differences observed in vine symptom severity could not be explained by either the virus accumulation or the composition of molecular variant number.

## 4. Discussion

Cross-protection aims to mitigate the impact of viral diseases through the use of plants pre-immunized with mild virus strain(s) to protect them from disease resulting from superinfections by more severe strain(s) of the same viral species. This approach is an attractive biocontrol method, especially for endemic viral diseases for which no management solutions are available [34]. The first and most salient need for this method to be effective in agricultural crops is the selection of mild strains that induce milder symptoms than severe virus strains together with limited impacts on the crop [37]. With the aim to develop cross-protection against fanleaf degeneration in grapevine, the main objective of our study was to survey heavily diseased vineyards and identify vines displaying little to no symptoms that may be infected with potentially mild GFLV isolates. Thus, we selected two highly symptomatic, 30-year-old, ‘Chardonnay’ vineyard parcels in the Champagne region of France and monitored eleven vines showing mild symptoms and ten severely symptomatic vines. Vines were selected based on visual symptoms assessed in springtime. Although visual symptom ratings are subjective, a PCA showed a robust relationship between qualitative phenotypic scores on disease symptoms in spring and quantitative traits recorded on fruits at harvest (Figure 2). Hence, the overall symptoms and the fruit yields were strongly negatively correlated (Table S4). Such association between severe GFLV symptoms and poor productive performance of the vine is consistent with results obtained in an experimental vineyard [13]. However, to our knowledge, it is the first time that viral symptomatology and agronomic performances were simultaneously considered in fanleaf-diseased commercial vineyards, illustrating a statistically supported negative correlation [9]. It will be interesting to confirm this result with a higher number of ‘Chardonnay’ vines and with other grapevine cultivars in other vine-growing regions.

Phenotypes and the GFLV infection status were stable from 2016 to 2019 for the three categories of vines identified (mild symptomatic GFLV-free vines (M−), mild symptomatic GFLV-infected vines (M+) and severe symptomatic GFLV-infected vines (S+)). In the category M−, seven vines remained free of GFLV (over the four years of the study) even though they were proximal to GFLV-infected vines. This may be due to an inefficient GFLV transmission by nematode vectors as a result of their well described aggregate population distribution in the field [61,62]. By comparing GFLV-infected with GFLV-free vines, GFLV always had a negative impact on fruit yield, reaching a 40% reduction on average for M+ vines but as high as 65% for the S+ category. This range of crop losses due to GFLV is in accordance with those previously described [9,13]. Given the interest of M+ category vines as candidates for cross-protection, a gain in production close to 82% in comparison to the S+ vines category was noted. This is in accordance with the performances reported in the literature for mild cross-protective viral strains, like zucchini yellow mosaic virus (ZYMV) strain WK on *Cucumis melo* (cv. TopMark) or PepMV strains Sp13 and PS5 on *Solanum lycopersicum* (cvs. Caniles and Pitenza) [40,63]. Moreover, the average fruit yield for vines in the M+ category over the four-year study (i.e., 1.8 kg per vine) is within the range of official vine production standards defined for appellation ‘Champagne’ in France, i.e., maximum 1.9 kg per vine per year [64,65]. These results validate the performance of these four mild-symptomatic GFLV-infected vines and the selection of their GFLV molecular variants as possible interesting candidates for cross-protection implementation in ‘Chardonnay’ in the Champagne region of France.

Another criterion for cross-protection that should be carefully examined is the eventual presence of other viruses in the infected host plant, potentially responsible for specific intra host virus-virus interactions that might affect the ability of the mild strain at protecting the crop [66,67]. This is particularly important for grapevine for which multi-infection is common generally displaying a complex virome in the field [20,68,69,70,71,72,73,74,75]. With our RNA-Seq data, we showed that several viroids and viruses were present in the studied vines samples. The grapevine background virome, composed of GRSPaV, HSVd, GYSVd-1, and some viruses of the *Tymoviridae* family (GFkV, GSyV1, GRVFV, GRGV), was found in the three vine categories (M–, M+ and S+). The etiological role of these viruses with no known vectors remains unclear [76]. In addition to this background virome, two other viruses were found in some vines in the S+ category that could potentially add onto the impact caused by GFLV: GPGV, associated with grapevine leaf mottling and deformation; and GLRaV-2, part of the leafroll disease [77,78]. Overall, the number of viruses and viroids detected per vines in this study ranged from three (vines Pa3 and Py13) to eight (vine Py17). Different combinations of these viruses as well as distinct variants of each species (data not shown) were found, thus giving a unique virome for each studied vine. Since RNA-Seq after a poly-A selection does not provide an exhaustive view of the virome, we cannot exclude that other viruses not detected using this protocol are present in the studied vines [19,79,80]. Furthermore, our results were obtained from leaf samples (which might be not fully representative of the entire plant), and it would be interesting to study further the grapevine virome and the distribution of sub-populations in different compartments of the vines. Current basic knowledge on the vine’s viruses does not allow for the anticipation of the impact of these mixed infections. In our data, it is interesting to mention that GFLV always accumulated more than any other co-infecting viruses in any of the vines, displaying higher RPKM.

Another objective of our work was to characterize the GFLV variants present in vines in the M+ and S+ categories to potentially identify molecular features specific to mild isolates. No correlation was found between the phenotypic category (M+/S+) and viral genomic RNAs accumulation when comparing RPKMs (Figure 7). This finding is consistent with previous studies describing no greater GFLV accumulation in ‘Gewurztraminer’ displaying severe stunting than in those showing mild symptoms [13]. Viral symptoms mostly arise from specific interactions between virus and host components rather than from high viral accumulation [14,81]. As mixed infection by distinct variants of a virus can be responsible for an increase in symptoms [82], we compared the number of molecular variants in vines of the M+ and S+ categories. No statistical difference was found between these two categories of vines (Figure 7). In some cases, viral satellite RNAs can be implicated in symptom development [83,84]. GFLV satellite RNA3 was absent in vines of the M+ category but was detected in four out of ten vines in the S+ category. From these data, we cannot conclude about a possible contribution of RNA3 in symptom modulation. The implication of GFLV RNA3 in severe disease symptom expression had been previously ruled out [22,85], but more work would be needed to definitely refute this hypothesis. Finally, by considering complete ORF1 and ORF2 sequences and sequences of the coding regions of these ORFs, no genetic differentiation due to genetic structure was observed between GFLV populations from vines in the M+ and S+ categories. However, some specific features of mild isolates might not be detected because of some limitations of our metagenomic approach. One limitation resides in the fact that multiple genetically divergent GFLV molecular variants were detected in most of the vines, and that the combination of GFLV ORF1 and ORF2 molecular variants were almost unique in each vine (Table 2). Thus, it would have been preferable either to focus on vines showing contrasted phenotypes and hosting a single variant of each genomic RNA (which seems rare in vineyards) or to study a higher number of vines for each phenotypic category to reveal specific genetic features of mild GFLV sequences. Another limitation deals with sequence comparisons performed on large genomic regions, thus providing little granularity to reach solid conclusions, knowing that a unique residue could cause a specific phenotype, as recently demonstrated for GFLV in the *N. benthamiana* model plant system [16]. As the GFLV symptom determinants are not yet known in grapevine, it was impossible to investigate in detail a specific region of the viral genome.

While no differences in GFLV populations between vines in the M+ and S+ categories could be determined, according to our *F_ST_* analysis (*p* < 0.05), a genetic differentiation according to Pa/Py vineyard parcels was observed for both ORF1 and ORF2 sequences (Figure 6). This appeared to be specific to GFLV since such genetic structuration was not shown for GRSPaV populations detected in vines from both parcels (*F_ST_*_(GRSPaV Pa/Py)_ = 0.002, *p* = 0.410 ± 0.014). It is the first time that genetic differentiation between vineyard sites within a same vine-growing region is described for GFLV. Indeed, while differential geographic structuration of GFLV populations between countries have been previously described [17,86], no genetic differentiation was observed between populations from three naturally GFLV-infected Californian vineyard sites [26]. Additionally, in France in 2004, the comparison of 85 sequences of the 2C^CP^ coding region of GFLV isolates from two vineyard parcels located 500 m apart in the Champagne region showed no genetic differentiation according to the parcel [18]. The genetic differentiation according to populations from the Pa and Py vineyard sites highlighted here might be explained by: (i) GFLV genetic distant variants brought into the Pa and Py vineyard sites by independent human activities such as the introduction of infected planting material or transfer of infested soil, and/or (ii) viral evolution selecting specific variants for best adaptation to each vineyard site.

A significant requirement for cross-protection is that the mild strain should protect against a broad range of severe strains [37]. Regarding GFLV, this feature appears essential as this virus exists as a population of numerous and sometimes unique (this study) genetically distant molecular variants within a vineyard site [18,20,26,55,87,88]. In the Pa and Py vineyards, the overall nucleotide sequence diversity (π) was similar to the one observed for GFLV sequences from all around the world either for ORF1 or ORF2 sequences [17]. As previously observed [17,18,55,88,89,90], intra-species recombination events were predicted in this study. Mixed infection with more than one genetically divergent molecular variant of each GFLV genomic RNA were found in most vines in the Pa and Py vineyard sites. Mixed infection can arise from simultaneous transmission of multiple variants of GFLV since a single *X. index* can retain more than one molecular variant of GFLV [20] or from successive inoculations over time by viruliferous *X. index* inoculating a single molecular variant. Considering the high genetic diversity of GFLV within a vineyard, it might be interesting to set up field experiments by using vines infected with several mild variants of GFLV for broad-spectrum cross-protection. Indeed, it has been demonstrated that superinfection exclusion is dependent on the genetic proximity between the protective strain and its challengers [36]. Strain-specific protection was clearly observed for CTV [91,92], ZYMV [43,93], and papaya ringspot virus (PRSV) [48,94]. In this context, the four mild symptomatic vines that host potentially mild GFLV isolates that were identified in our study might be of great interest for cross-protection, as they belong not only to divergent phylogenetic lineages but also to the two most represented clades of GFLV ORF1 and ORF2 (sequences clades I.A, I.B, II.A and II.B), and were detected in both sites except for clade I.A. Moreover, two mild variants of two divergent strains of PepMV were more effective at protecting tomato plants against divergent aggressive variants of these strains than a unique mild strain [40]. Similar to the mixture of molecular variants identified in the PIAC mild CTV isolate [34,95], coinfection by two genetically distant variants of GFLV RNA1 was detected in vines Py18 and Py19. It will be interesting to compare if GFLV isolates from the M+ category from vines Py18 or Py19 will be more efficient at protect against a broad range of severe isolates than GFLV isolates from vines Pa1 and Py43. Similarly, it would be interesting to know if protection is as effective for the two genomic RNAs of the virus with regard to the presence of multiple divergent molecular variants.

## 5. Conclusions

In summary, our results revealed that: (i) vines displaying mild symptoms were identified in a heavily fanleaf diseased vineyard; (ii) these plants were infected with potential GFLV mild isolates (need to be further tested); (iii) no molecular features specific to GFLV variants present in these mild symptomatic vines were detected and iv) for the first time a genetic differentiation of GFLV populations between two parcels from the same viticultural region was detected.

## Figures and Tables

**Table 1:** Presence (+) or absence (−) of grapevine fanleaf virus (GFLV) in leaves of individual ‘Chardonnay’ vines collected in spring from 2016 to 2019, as determined by DAS-ELISA. Vines were classified into three categories: M− (mild symptomatic, GFLV-free vines), M+ (mild symptomatic, GFLV-infected vines) and S+ (severe symptomatic, GFLV-infected vines). The presence/absence of GFLV in vine Py43 was only tested starting in 2017 as this plant was only identified in autumn 2016 (nd: not determined).

	M−							M+				S+									
	Pa3	Pa5	Pa7	Pa9	Py11	Py13	Py16	Pa1	Py18	Py19	Py43	Pa2	Pa4	Pa6	Pa8	Pa10	Py12	Py14	Py15	Py17	Py20
**2016**	−	−	−	−	−	−	−	+	+	+	nd	+	+	+	+	+	+	+	+	+	+
**2017**	−	−	−	−	−	−	−	+	+	+	+	+	+	+	+	+	+	+	+	+	+
**2018**	−	−	−	−	−	−	−	+	+	+	+	+	+	+	+	+	+	+	+	+	+
**2019**	−	−	−	−	−	−	−	+	+	+	+	+	+	+	+	+	+	+	+	+	+

**Table 2:** Molecular variants composition of grapevine fanleaf virus in individual ‘Chardonnay’ vines and their classification in phylogenetic clades (Figure 5). The clade names are ‘I’ for ORF1, ‘II’ for ORF2 and ‘III’ for ORF3. The number of molecular variants is indicated. When two molecular variants affiliated to a same clade are present in a vine, their numbers are separated by a slash.

	M+				S+									
	Pa1	Py18	Py19	Py43	Pa2	Pa4	Pa6	Pa8	Pa10	Py12	Py14	Py15	Py17	Py20
**I.A**				1-1						1-1/1-2	1-1/1-2	1-1		1-1
**I.B**		1-1	1-1						1-2		1-3		1-1	
**I.C**					1-1	1-1	1-1							
**I.D**										1-3		1-2		
**I.E**								1-1	1-1					
**I.F**														1-2/1-3
**I.G**					1-2									
**I.H**		1-2												
**I.I**			1-2											
**I.J**							1-2							
**I.K**													1-2	
**I.L**	1-1													
**II.A**			2-1			2-1					2-2		2-2	2-1
**II.B**	2-1	2-1							2-2	2-2		2-2		
**II.C**				2-1						2-1	2-1	2-1	2-1	
**II.D**								2-1	2-1					
**II.E**					2-1									
**II.F**							2-1							
**III.A**					3-1/3-2	3-1	3-1							
**III.B**													3-1	

## Data Availability

RNA-Seq data has been deposited in the ENA-EBI, project N°PRJEB54883.

## Supplementary Materials

The following supporting information can be downloaded at: https://www.mdpi.com/article/10.3390/v14102303/s1, Figure S1: Schematic representation of the location of the selected vines in vineyard sites ‘Pa’ and ‘Py’; Figure S2: Disease symptom scoring; Figure S3: Comparison of phenotypes and fruit yields of ‘Chardonnay’ vines in the M and S classes in 2016; Figure S4: Mean symptom scores and fruit yields of ‘Chardonnay’ vines in the M−, M+ and S+ categories from 2016 to 2019; Figure S5: Comparison of symptoms scores and fruit yields of ‘Chardonnay’ vines over four years; Figure S6: Intraspecies recombination events in the grapevine fanleaf virus (GFLV) ORF1 and ORF2 sequences from vines in the ‘Pa’ and ‘Py’ vineyard sites; Table S1: Raw data of qualitative and quantitative traits monitored over four years (2016–2019) on the selected grapevines; Table S2: Results of Student’s *t* test, Welch’s *t* test or Kruskal–Wallis *H* test assessing the symptom class (S/M) effect on qualitative and quantitative traits; Table S3: Results of Tukey HSD post hoc test on different categories for all the variables measured from 2016 to 2019 and over four years; Table S4: Results of Fisher’s test assessing the correlations between all the variables and the first principal component (PC1); Table S5: Reads per kilobase per million reads (RPKM) measured for each virus and GFLV molecular variants de novo assembled from vines in the ‘Pa’ and ‘Py’ vineyard sites; Table S6: Nucleotide and amino acid sequence identity matrix between GFLV ORFs sequences; Table S7: Genetic diversity analyses of sequences in the coding regions of GFLV ORF1 and ORF2; Table S8: Results of Student’s *t* or Kruskal–Wallis *H* tests assessing the phenotypic category (S+/M+) or the vineyard origin (Pa/Py) effect on RPKM values or number of variant for each genomic RNA.
