# Supplementary material for: Characterization of Grapevine Fanleaf Virus Isolates in ‘Chardonnay’ Vines Exhibiting Severe and Mild Symptoms in Two Vineyards
Source: Viruses. 2022 Oct 20;14(10):2303. doi: 10.3390/v14102303 (PMC9609649; doi:10.3390/v14102303)
Supplement: Supplementary file 1 [file viruses-14-02303-s001.zip › 2022_Kubina_Viruses_Supplementary Figures_Proof.pdf]

Supplementary Figure S1. Schematic representation of the location of the selected vines in vineyard sites 'Pa' (A) and 'Py' (B, C, and D). Site 'Pa' (A) is a 1.4 hectare parcel composed of two separate blocks: four vines were selected in one block and six vines in the other block. Some selected vines were proximal to each other (the between-row distance was about 1.5 meter), others were about 230 meters apart. Site 'Py' (B) is a 3.35 hectare parcel with vines either in close proximity or 40 meters apart. Pictures of some of the selected vines in site 'Py' site were taken in June 2018 (C) and June 2019 (D).

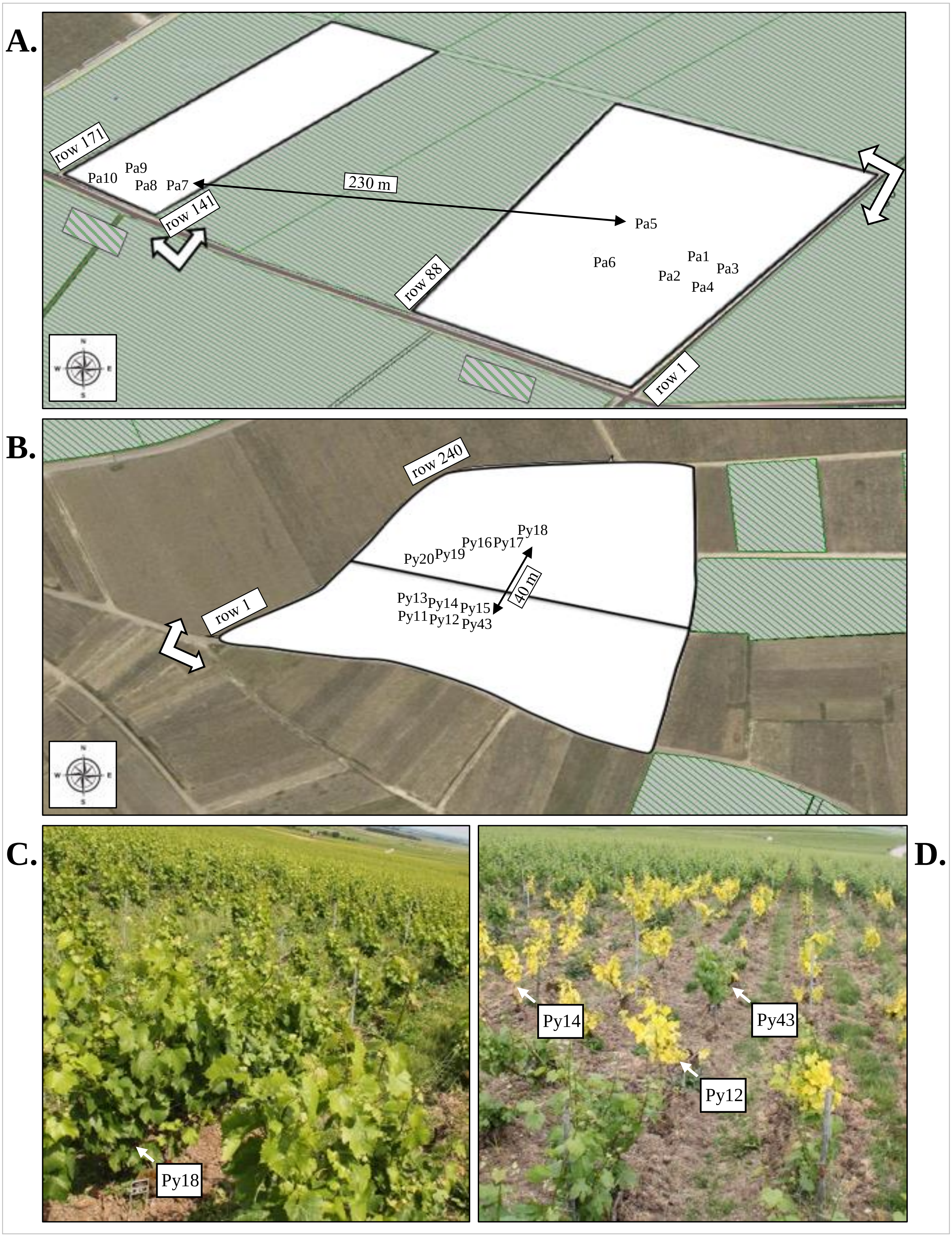

Supplementary Figure S2. Disease symptom scoring. Examples of qualitative phenotypic traits from the ‘Pa’ and ‘Py’ vineyard sites in 2016 and 2017. (A) From left to right: leaf discolourations (yellowing, vein banding, mosaic) and deformations (asymetric blades, small leaves with open petiolar sinus). (B) Leaf yellowing scores from 0 to 4 (from left to right, 0 with no visible symptoms, 1 with 1-25%, 2 with 26-50%, 3 with 51-75% and 4 with 76-100% of the leaves symptomatic).

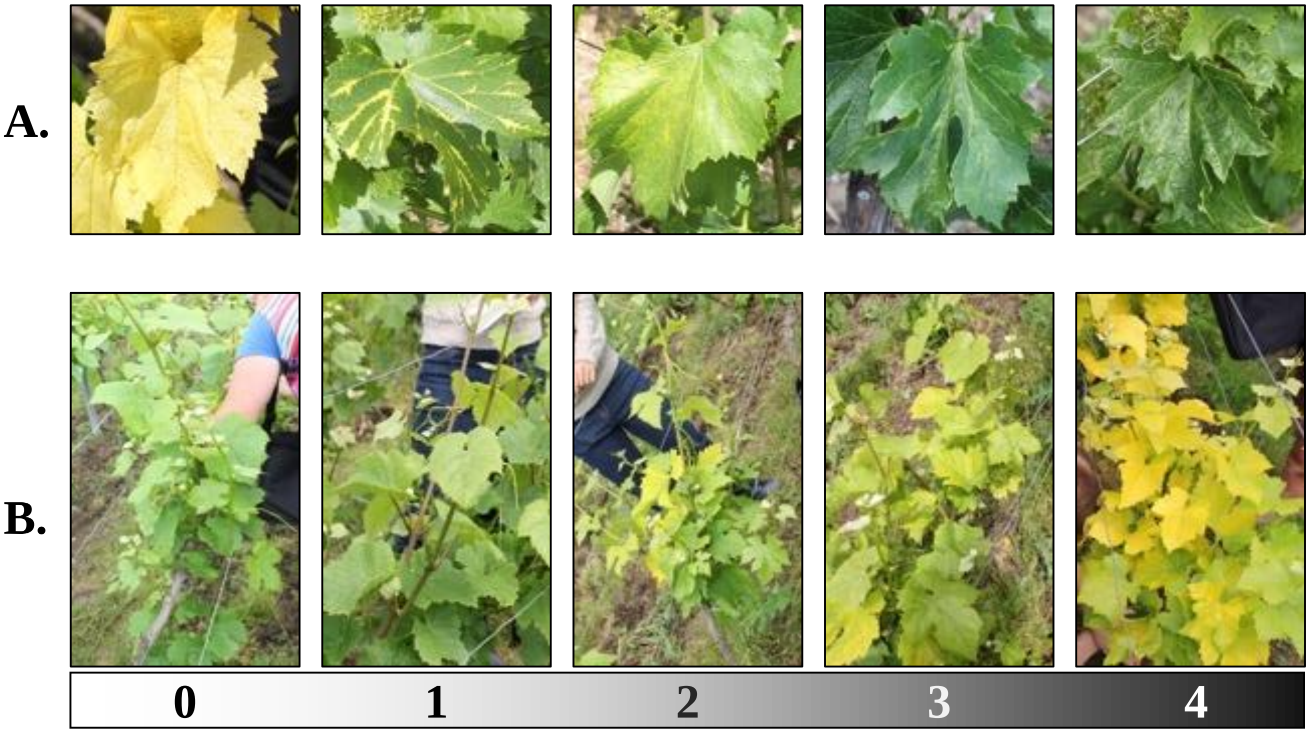

Supplementary Figure S3. Comparison of phenotypes and fruit yields of 'Chardonnay' vines in the M and S classes in 2016. Qualitative and quantitative phenotypic traits were compared for the two classes of vines: mild symptomatic vines (M, in white) and severely symptomatic vines (S, in black). Number of vines (n) is given in brackets. *For the M class, only 10 vines were considered in June 2016 for the evaluation of the qualitative traits (leaf discoloration, leaf deformation, vine stunting). Boxplots show the median (horizontal bold line) and the interquartile range with lonely dots representing extreme data. Significance was tested with Student's *t*, Welch's *t* or Kruskal-Wallis *H* tests. Different letters (a,b) indicate significant differences ($P \leq 0.05$). Raw data and results of the statistical tests are available in **Supplementary Tables 1 and 2,** respectively.

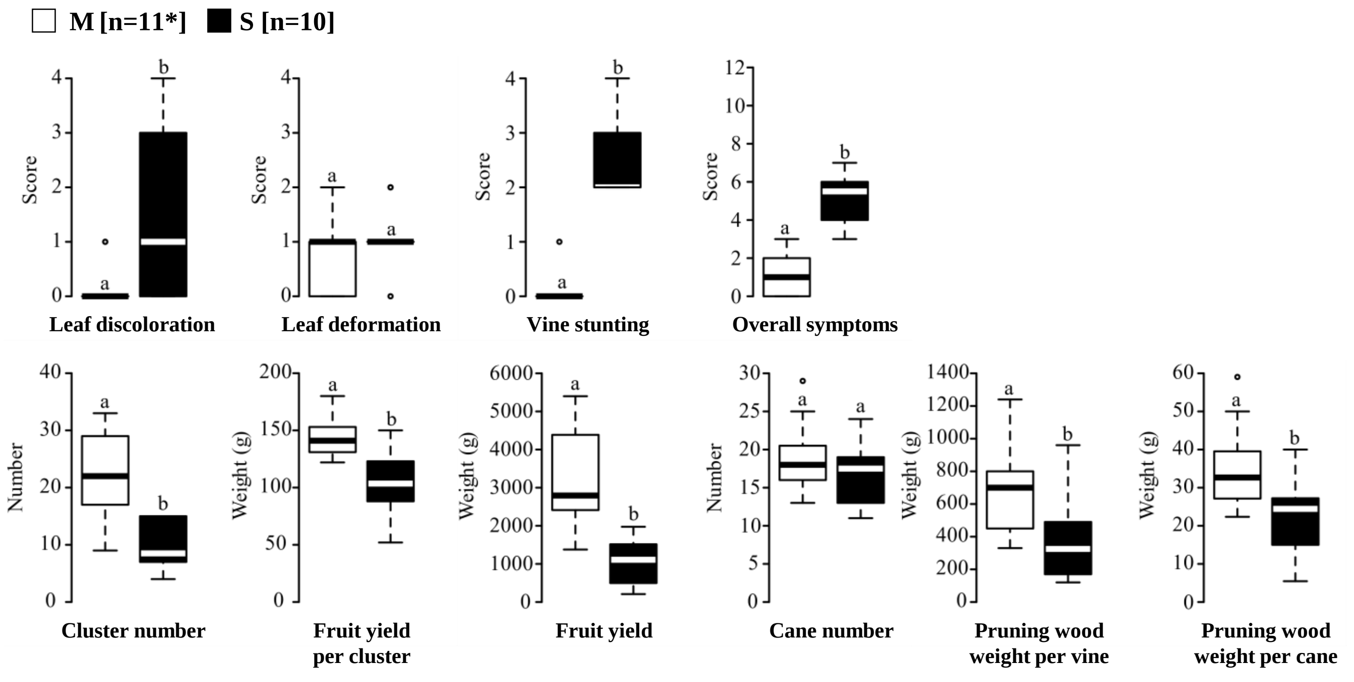

Supplementary Figure S4. Mean symptom scores and fruit yields of ‘Chardonnay’ vines in the M-, M+ and S+ categories from 2016 to 2019. Results obtained with vines in the M− (in grey), M+ (in cyan) and S+ (in black) categories are shown. Number of vines (n) is given in brackets. For clarity, means have been connected by bold lines and standard errors materialized by vertical bars. *For the M+ category, only three vines were considered in June 2016 for the evaluation of qualitative traits (leaf discoloration, leaf deformation, vine stunting).

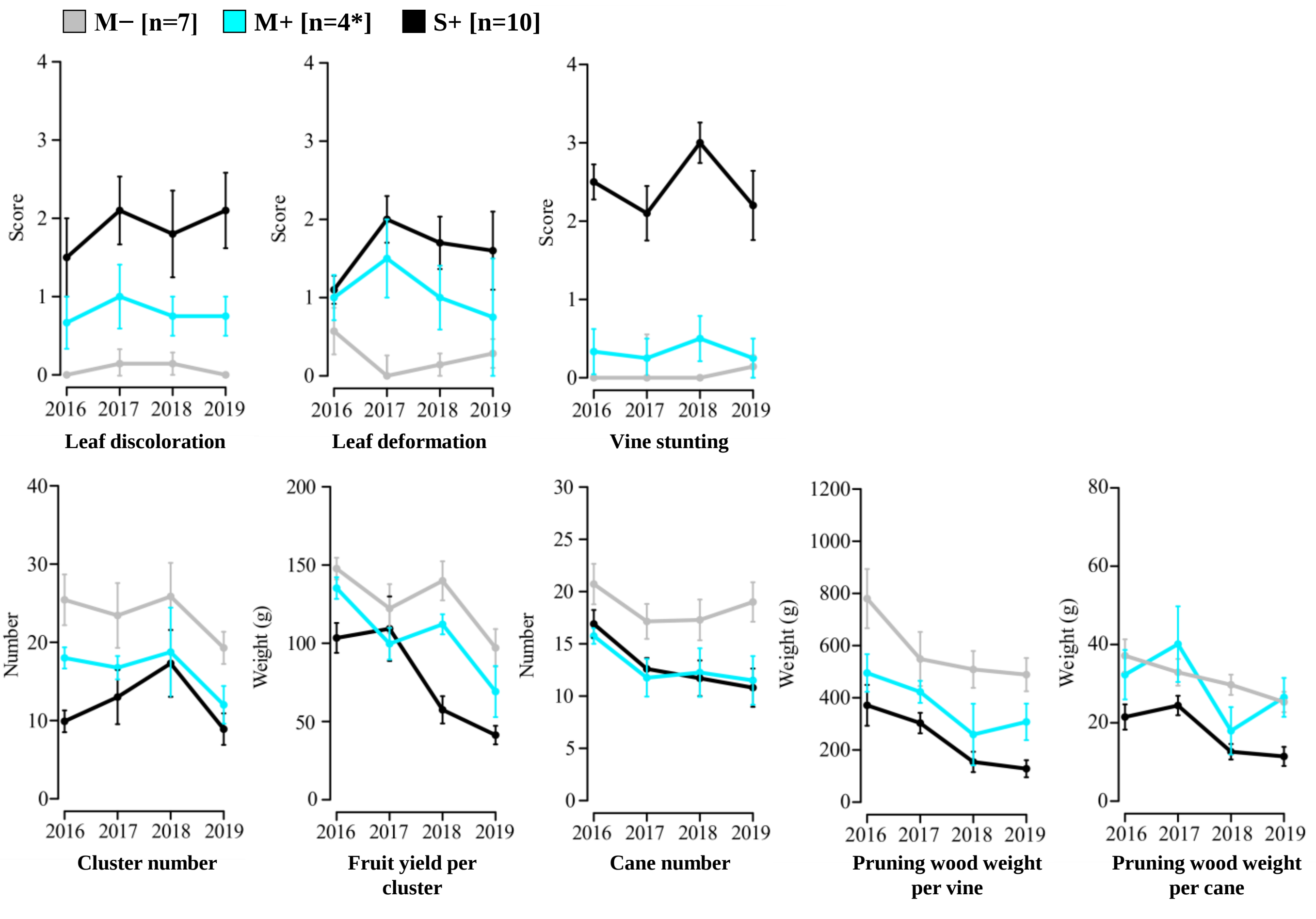

Supplementary Figure S5. Comparison of symptoms scores and fruit yields of 'Chardonnay' vines over four years. Results obtained with vines in the M− (in grey), M+ (in cyan) and S+ (in black) categories are shown. Boxplots show the median (horizontal bold line) and the interquartile range with lonely dots representing extreme data. Different letters (a,b) indicate significant differences ($P < 0.05$). Raw data and results of the statistical tests are available in **Supplementary Tables 1** and **3,** respectively. Number of replicates (n) is given in brackets. *****For the M+ category, 15 measurements were considered for the evaluation of qualitative traits (leaf discoloration, leaf deformation, vine stunting). **In 2017, vine Py14 did not produce any fruits, thus decreasing to 39 the number of replicates in the S+ category for the calculation of fruit yield per cluster.

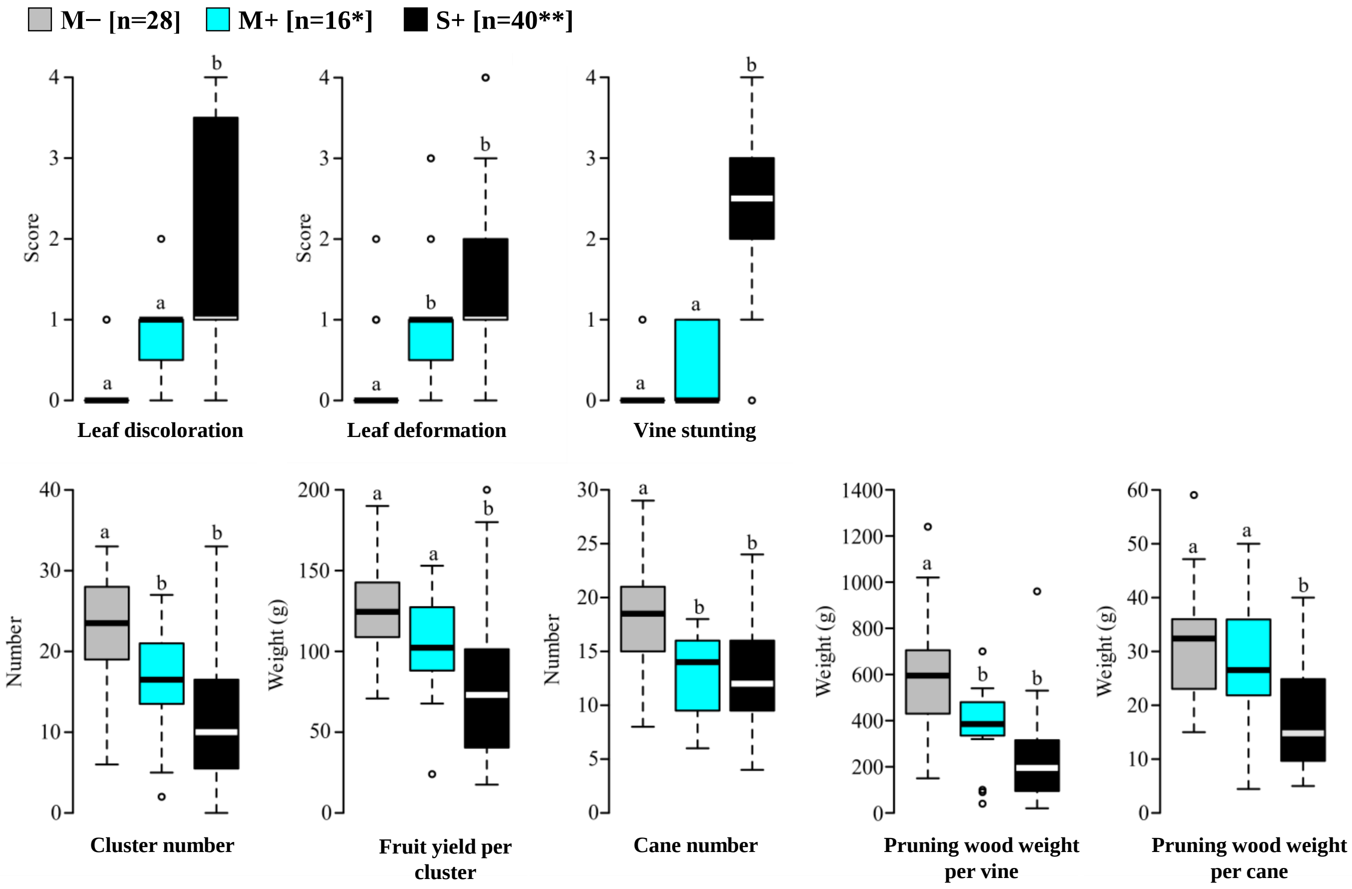

Supplementary Figure S6. Intraspecies recombination events in the grapevine fanleaf virus (GFLV) ORF1 (A) and ORF2 (B) sequences from vines in the 'Pa' and 'Py' vineyard sites. Location of recombination sites detected in ORF1 and ORF2 sequences by Recombination Detection Program package 4 (RDPv.4.46), with RDP (R), GeneConv (G), BootScan (B), MaxChi (M), Chimaera (C), Siscan (S) and 3Seq (3) softwares. Only recombination events detected by five or more methods were considered. Schematic representation along ORFs nucleotide sequences is given with the number of the recombination event indicated within circle. Details and precise locations of recombination events, major and minor parents as well as P-values are indicated in the tables below each genomic RNAs.

A.

GFLV RNA1

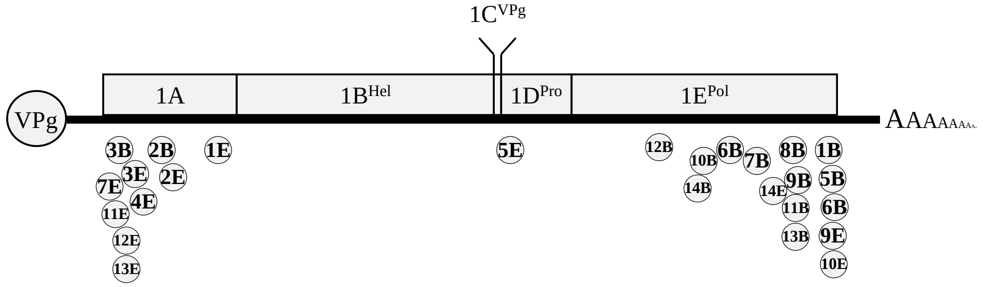

recombinant		breaking point location		parents				RDP4	
N°	name	beginning (B)	end (E)	major	% sim	minor	% sim	corrected *P*	software
1	Py12-1-2	6844 [6817-16]	1065 [1015-1083]	Py14-1-1	99.7	Py15-1-2	99.9	6.25^{E-25}	R.G.B.M.C.S.3
2	Pa6-1-2	546 [476-599]	655 [660-881]	Pa6-1-1	88	Py18-1-2	99.1	1.10^{E-5}	G.B.M.C.3
3	Pa1-1-1	132 [103-162]	297 [206-422]	Py15-1-1	89.2	Pa6-1-1	97	7.07^{E-9}	R.G.M.C.3
4	Py18-1-2	undetermined	381 [231-872]	Pa6-1-1	undetermined	Py18-1-1	97.8	3.03^{E-5}	R.G.B.M.S.3
5	Py17-1-2	6846 [6823-361]	3814 [3761-3917]	Py18-1-2	96.8	Py17-1-1	98.5	5.12^{E-63}	R.G.B.M.C.S.3
6	Py20-1-3	5865 [5836-5885]	6844 [6832-29]	Py20-1-2	100	Py20-1-1	100	6.136^{E-113}	G.B.M.C.S.3
7	Pa1-1-1	6126 [3274-6197]	58 [6827-138]	Pa8-1-1	undetermined	Py19-1-2	94.4	1.31^{E-22}	R.G.B.M.C.S.3
8	Py19-1-2	6452 [5479-6194]	undetermined [6814-1895]	Pa10-161	undetermined	Pa6-1-2	92.2	2.64^{E-16}	R.G.B.M.C.S.3
9	Py18-1-2	6504 [6310-6564]	6825 [6792-8]	Pa8-1-1	89.9	Py19-1-1	undetermined	4.84^{E-12}	R.G.B.M.C.S.3
10	Pa6-1-1	5617 undetermined	6844 [6784-229]	Py14-1-3	undetermined	Py15-1-1	91.6	9.37^{E-10}	R.G.B.M.C.S.3
11	Py19-1-1	6480 [6337-6532]	112 [6790-217]	Pa10-1-1	89.1	Py20-1-2	undetermined	1.72^{E-8}	R.M.C.S
12	Pa10-1-1	5206 [3448-6526]	214 [6815-1832]	Py15-1-1	undetermined	Py20-1-2	89.1	1.78^{E-8}	R.M.C.S.3
13	Pa2-1-2	6476 [5347-6507]	214 [6814-1637]	Py20-1-2	89	Py15-1-1	undetermined	1.02^{E-6}	R.M.C.S
14	Py19-1-2	5562 [5464-5792]	6270 [6166-1841]	Py14-1-2	undetermined	Pa6-1-2	93.4	7.13^{E-6}	R.B.M.C.S.3

B.

GFLV RNA2

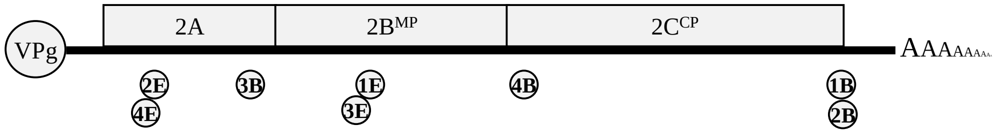

recombinant		breaking point location		parents				RDP4	
N°	name	beginning (B)	end (E)	major	% sim	minor	% sim	corrected *P*	software
1	Py15-2-2	3306 [3209-121]	1194 [1133-1215]	Pa1-2-1	98.5	Py19-2-1	98.1	2.15^{E-12}	G.B.M.C.S.3
2	Py19-2-1	3315 [3205-50]	226 [166-242]	Py20-2-1	97.3	Py18-2-1	98.8	8.15^{E-8}	R.G.B.M.3
3	Pa2-2-1	656 [619-677]	1128 [1075-1148]	Py18-2-1	98.3	Py20-2-1	98.5	4.72^{E-12}	G.B.M.C.S.3
4	Pa2-2-1	1881 [1825-191]	188 [148-227]	Pa10-2-2	95.7	Pa6-2-1	99	2.87^{E-25}	G.B.M.C.S.3
